# The Influence of Patient‐, Site‐, and Implant‐Related Factors on Marginal Bone Levels of Dental Implants in a Rural Population in China: A Retrospective Study

**DOI:** 10.1002/cre2.70045

**Published:** 2024-11-22

**Authors:** Anahat Khehra, Ossi Zanner, Nachum Samet, Liran Levin

**Affiliations:** ^1^ Division of Periodontology, Department of Oral Medicine, Infection and Immunity Harvard School of Dental Medicine Boston Massachusetts USA; ^2^ One‐Stop Dental Hospital Beijing China; ^3^ Private Clinic Tel Aviv Israel; ^4^ College of Dentistry University of Saskatchewan Saskatoon SK Canada

**Keywords:** implant survival, peri‐implant health, peri‐implantitis, success

## Abstract

**Objectives:**

Limited research is available on implant treatment outcomes in rural populations. This may be due to the presence of various barriers, such as access to oral health care, resources, health literacy, and education. The aim of this study was to evaluate the influence of patient‐, site‐, and implant‐related factors on marginal bone levels of dental implants in a rural population in China.

**Material and Methods:**

A retrospective study was conducted using data from a private dental office. Subjects included in this study received dental implants as part of their routine dental treatment. Information on age, gender, smoking status, diabetes, heart disease, jaw location, restorative type, loading protocol, survival rate, implant length, and diameter was collected. Marginal bone loss was recorded as the largest value at either the mesial or distal aspect on peri‐apical radiographs. Descriptive and inferential statistics were performed along with linear regression analysis.

**Results:**

Overall, 428 implants were placed in 90 subjects over an average follow‐up period of 453 days. No implant failures were recorded. The average marginal bone loss was 0.10 mm, with 80.6% of implants showing no marginal bone loss. The extent of marginal bone loss was greater in the mandible (0.13 ± 0.25) than in the maxilla (0.08 ± 0.19). An increase in implant diameter by 1 mm resulted in 0.08 mm of marginal bone loss, indicating wider diameter implants are associated with more bone loss. Age was also positively correlated with marginal bone loss, increasing by 0.002 mm per year. No differences were found for gender, smoking, diabetes, heart disease, restoration type, and immediate loading.

**Conclusions:**

Dental implant therapy in a rural Chinese population demonstrated high survival rates and minimal marginal bone loss. Factors such as age, implant location, and diameter influenced bone loss. This study fills a critical gap in understanding implant outcomes specifically within rural settings, highlighting the need for tailored approaches to enhance patient access and care in these communities. Further research is needed to explore these relationships and assess implant outcomes in rural populations.

## 1 Introduction

Marginal bone loss around dental implants may create an environment more favorable for disease progression (Nevins et al. 2013). A multitude of causes may contribute to marginal bone loss, including surgical trauma, excessive occlusal forces, systemic factors, peri‐implant disease, prosthetic design, and implant micro‐ and macro‐design (Oh et al. 2002; Canullo et al. 2016; Schliephake 2022; Schwarz et al. 2018; Hamilton et al. 2023; De Bruyn et al. 2017). To mitigate the risk of marginal bone loss, a careful examination and well‐thought‐out surgical protocol must be completed. Understanding the local and systemic factors that may influence treatment outcomes is important (Schliephake 2022), as it assists in the planning, execution, and maintenance of dental implant therapy. Following surgical intervention, it is crucial to establish proper oral hygiene techniques and concordance with maintenance appointments (Cortellini et al. 2019; Kwon et al. 2020; Clark and Levin 2016). Early detection of disease allows for the delivery of appropriate care and prevention of marginal bone loss (Kwon, Yen, and Levin 2022).

Certain factors, such as prosthetic design and implant micro‐ and macro‐design, vary and may influence bone level stability around dental implants (Hamilton et al. 2023; De Bruyn et al. 2017). The type of restoration (cement or screw retained), contour of restoration, and implant–abutment connection are a few aspects of the prosthetic design that must be carefully evaluated (Lemos, de Souza Batista, Almeida, Santiago Júnior, et al. 2016; Katafuchi et al. 2018; Caricasulo et al. 2018). A consistent point of discussion is the implant–abutment connection. Many studies have outlined that an internal or conical connection is associated with less peri‐implant bone loss compared to an external connection (Caricasulo et al. 2018; Koo et al. 2012; Peñarrocha‐Diago et al. 2013; Pozzi et al. 2014). In vivo and in vitro studies have demonstrated that an external connection carries a high risk of bacterial contamination (Koutouzis, 2019). As a result, dental professionals may favor the use of internal connection implants over external connection types.

Currently, the role of immediate versus delayed implant loading is of clinical and research interest. A 1‐year randomized controlled trial found that mean marginal bone loss was 0.83 mm in the immediate loading group and 0.86 mm in the delayed loading group, with no statistically significant difference (Meloni et al. 2012). Similarly, a systematic review and meta‐analysis found that there is no statistically significant difference in survival rates and marginal bone loss after 5 years of function (Benic, Mir‐Mari, and Hämmerle 2014a). Regardless of loading time, it is essential to evaluate occlusion to ensure that no excessive forces or interferences are present (Sheridan et al. 2016).

It is also crucial to understand that there are various surface modifications available that can change implant micro‐design, thus influencing osseointegration and marginal bone loss (Kligman et al. 2021). Machining, sandblasting, laser‐etching, acid‐etching, anodizing, plasma spraying, and calcium phosphate coating are a few examples of surface treatments. In general, roughened surfaces improve osseointegration, reduce marginal bone loss, and increase survival rates compared to machined, smooth surfaces (Kligman et al. 2021; Zhang and Yue 2021; Messias, Nicolau, and Guerra 2019). However, once exposed, a rough surface is more likely to harbor bacteria, thereby increasing the risk of peri‐implant disease (De Bruyn et al. 2017). Hence, it is important to perform primary prevention measures before, during, and after implant placement to avoid the consequences of marginal bone loss and exposure.

Many different implant macro‐designs are available on the market. The implant shape, thread pattern, diameter, and length should be thoroughly evaluated, as each aspect may influence treatment outcomes (Atieh, Alsabeeha, and Duncan 2018; Abuhussein et al. 2010; Renouard and Nisand 2006). Atieh, Alsabeeha, and Duncan reported in a systematic review and meta‐analysis that tapered implants have higher primary stability and less marginal bone loss, but no difference in failure rate compared to parallel‐walled implants (Atieh, Alsabeeha, and Duncan 2018). Potential addition of threads to the crestal third of the implant may increase bone‐to‐implant contact and preserve marginal bone; however, further studies are needed to verify the presence of association (Abuhussein et al. 2010). Conflicting evidence exists on the impact of implant length and diameter on treatment outcomes. Renouard and Nisand conducted a literature search and found that short implants had a similar or increased failure rate to longer ones, and wide‐diameter implants have no relationship with increased failure (Renouard and Nisand 2006). Another study reported that wide, long, and untapered implants minimized crestal bone strain, thereby decreasing the risk of bone loss (Petrie and Williams 2005). However, some of these studies used older dental implant systems, and more recent data are required to draw conclusions on the newer systems.

The majority of studies investigating success and survival criteria for dental implants include patients from urban areas. This may be due to barriers in accessing oral health care in rural settings (Khan, Thapa, and Zhang 2017; Luo et al. 2020). Park et al. found that tooth loss is prevalent in an elderly rural population, yet awareness and education on implant therapy are lacking (Park et al. 2017). As a result, few studies exist to assess peri‐implant health and outcomes in rural populations.

Overall, there are numerous factors to consider in the prevention and management of peri‐implant bone loss. It is vital for the surgeon to have a comprehensive understanding of the implant system being used to achieve favorable outcomes. A thorough clinical and radiographic assessment of the surgical site allows for careful implant selection for esthetics and function. In recent years, with the economic changes in China during the last decade, more and more patient populations have the financial ability to afford modern dental treatments, including dental implants. The aim of this study was to evaluate the influence of patient‐, site‐, and implant‐related factors on marginal bone levels of dental implants in a rural population in China.

## 2 Methods

A retrospective study was conducted using data from a private dental office in rural China. The surgical procedures were carried out by a team of seven clinicians. The measurements and information extracted for the study were performed by a team of researchers. The inclusion criteria consisted of subjects who received dental implants. The exclusion criteria consisted of subjects who had a history of a previously failed implant at the site of interest, active periodontal disease, uncontrolled medical conditions, and nursing or pregnant women.

The following baseline information was collected: (1) patient's age at the time of implant placement, (2) gender, (3) smoking status, (4) presence or absence of diabetes mellitus, and (5) history of heart disease. In addition, the location of implant placement (maxilla or mandible) was noted. Implant characteristics, such as length, diameter, and cement or screw‐retained restoration type, were also recorded. If bone augmentation was required during implant placement, this information was included in the data analysis along with the biomaterials used. The occurrence of immediate loading was also assessed as a factor.

Outcomes assessed following implant placement were survival and marginal bone loss. Bone loss was measured radiographically over time with documented follow‐up. A peri‐apical radiograph was used to assess crestal bone level at each follow‐up and identify changes as marginal bone loss. The implant length was used as a reference to determine the distance from the implant shoulder to bone‐to‐implant contact (French, Larjava, and Ofec 2015; Buser et al. 2012). The crestal bone level was then determined by subtracting the implant neck length from the distance between the implant shoulder and bone‐to‐implant contact. A single measurement was recorded at either the mesial or distal site, based on the largest value calculated.

A thorough evaluation of the data was conducted using descriptive statistics. A comparative examination using independent *t‐*tests and one‐way analysis of variance was also performed to investigate differences in gender, smoking, diabetes, heart disease, jaw type, implant restoration type, bone graft, membrane use, immediate loading, and marginal bone loss. To predict marginal bone loss for each independent variable, linear regression analysis was used. A *p‐*value of less than 0.05 was considered statistically significant.

## 3 Results

A total of 90 subjects were included in the study, and 428 implants (Touareg‐OS, Adin Dental Implant Systems, Alon Tavor, Israel) were placed (Table 1). The average subject age was 62 years, with a range from 28 to 88 years. Of the subjects, 58.6% were male, and 41.4% were female. The majority were non‐smokers (90.9%), non‐diabetic (74.8%), and had no history of heart disease (81.5%). Overall, 57.0% of the implants were placed in the maxilla.

**Table 1:** Descriptive statistics of variables: Age, implant length, implant diameter, marginal bone loss, and follow‐up days.

	Number	Minimum	Maximum	Mean	Standard deviation
Age	90	28	88	62.38	9.990
Length	428	8.0	18.0	13.044	1.9328
Diameter	428	3.50	5.00	3.87	0.35
Marginal bone loss	428	0.000	0.900	0.10234	0.224483
Follow‐up days	427	342	637	453.06	44.687

The average length and diameter of implants placed were 13 and 4 mm, respectively; 86.4% were screw‐retained, and 13.6% were cement‐retained. At the time of implant placement, bone augmentation was performed in 16.8% of subjects, and a membrane was used in 16.4%. Most implants were immediately loaded with a screw‐retained restoration (88.1%). The average follow‐up time was 453 days and ranged from 342 to 637 days. No implant failure was recorded during the follow‐up time. Marginal bone loss was not seen in 80.6% of implants, 0–0.5 mm loss occurred in 12.6%, and > 0.5 mm loss occurred in 6.8%. On average, marginal bone loss was 0.10 mm (Table 1).

No differences between groups were identified for gender, smoking, diabetes, heart disease, restoration type, and immediate loading. The placement of the implant in the maxilla and mandible had significant findings in relation to the length of the implant and the amount of marginal bone loss. The length of the implant was higher in the maxilla (13.3 ± 1.8) than in the mandible (12.8 ± 2.1). The extent of marginal bone loss was greater in the mandible (0.13 ± 0.25) than in the maxilla (0.08 ± 0.19).

The length of the implant was also found to be significantly longer for subjects who received bone augmentation (13.2 ± 2.0) compared to those who did not undergo augmentation (12.4 ± 1.6). In addition, the length was longer without a membrane (13.1 ± 2.0) than with one (12.4 ± 1.6).

Implant diameter was found to influence the extent of marginal bone loss. The wider the diameter, the more bone loss. Bone loss of > 0.5 mm (5.53 ± 8.53) was associated with larger diameter implants compared to those with no bone loss (3.97 ± 2.55). No difference was noted between no bone loss versus 0–0.5 mm of bone loss, or between 0 and 0.5 mm of bone loss and > 0.5 mm of bone loss.

A prediction of marginal bone loss influenced by each variable is depicted in Table 2. Linear regression analysis shows that age, jaw, and diameter of the implant may influence marginal bone loss. With each increase in year of age, marginal bone loss increased by 0.002 mm. In the mandible, marginal bone loss was 0.062 mm greater compared to the maxilla. An increase in implant diameter by 1 mm resulted in 0.08 mm of marginal bone loss.

**Table 2:** Association of marginal bone loss by each independent variable through linear regression analysis.

Variable	Significance (*p*‐value)	*R* ^2^	
Restoration type	0.819		
Age	**0.041**	1%	As age increases, marginal bone loss is greater. With each year, marginal bone loss increases by 0.002 mm.
Gender	0.566		
Smoking	0.497		
Diabetes	0.190		
Heart disease	0.145		
Jaw	**0.005**	1.9%	A greater marginal bone loss is seen in the maxilla than in the mandible by 0.062 mm.
Length	0.793		
Diameter	**0.009**	1.6%	The larger the diameter, the higher the bone loss. Every 1 mm increase in diameter results in 0.08 mm of marginal bone loss.
Membrane	0.439		
Immediate loading	0.604		
Follow‐up days	0.732		

*Note:* Bold numbers represent statistically significant values.

## 4 Discussion

A limited number of studies are available investigating the outcomes of implant therapy in rural populations. This may be due to the presence of oral health inequities, such as access to dental care, qualified professionals, and resources (Theriault and Bridge 2023). The implementation of tele‐dentistry, dental outreach groups, and incentives for practitioners could address geographical challenges (Nash, Nagel, and Conry 2008; Skillman et al. 2010). In addition, an effort to improve the distribution of information on the importance of prevention, timely treatment, and rehabilitation should occur. From here, investigation on the impact of treatment on rural populations can be recorded and assessed to provide recommendations on optimizing the delivery of care. This study aimed to evaluate factors influencing implant marginal bone in a rural population in China.

At the patient level, no differences in gender, smoking, diabetes, and heart disease were found between groups. However, age was shown to influence marginal bone loss. A 0.002 mm loss was noted with each additional year of age. Similarly, Negri et al. found a correlation between age and marginal bone loss in urban populations (Negri et al. 2014). Further assessment revealed that marginal bone loss progressively increased with age in male patients. Pedro et al. also reported that male gender and smoking were significantly associated with bone loss in urban populations (Ramos et al. 2017). However, age alone had no influence on bone loss and presented no contraindication to implant therapy. In this study, systemic diseases were not associated with marginal bone loss. It is important to consider the status of the systemic disease before proceeding with implant therapy. Javed and Romanos conducted a systematic review investigating the influence of glycemic control on osseointegration (Javed and Romanos 2009). It was determined that successful outcomes can be achieved in patients with good metabolic control; however, implant therapy should be considered a contraindication in patients with poor metabolic control. A thorough medical history must be obtained along with a careful assessment of the surgical site.

At the site level, no differences in restoration type and immediate loading were found between groups. Conflicting evidence on the influence of restoration type on marginal bone loss exists. Few studies indicate that there is no difference in marginal bone loss between cement and screw‐retained restorations (Wolfart et al. 2021; De Brandão, Vettore, and Vidigal Júnior 2013). In contrast, Lemos et al. reported that cement‐retained restorations showed less marginal bone loss, fewer prosthetic complications, and higher implant survival rates (Lemos, de Souza Batista, Almeida, et al. 2016). The influence of immediate versus conventional loading has also been investigated. Systematic reviews and meta‐analyses have revealed that implant survival and marginal bone loss are similar between the loading protocols (Chrcanovic, Albrektsson, and Wennerberg 2014; Benic, Mir‐Mari, and Hämmerle 2014b). In this study, jaw location was shown to be an influencing factor in marginal bone loss. An increased loss of 0.062 mm was found in the mandible compared to the maxilla. Eskandarloo et al. investigated the association between marginal bone loss and bone quality to find no significant differences (Eskandarloo et al. 2019). However, there was a correlation between higher bone quality and less marginal bone loss. Understanding the type of bone quality in the maxilla versus mandible and anterior versus posterior may impact implant survival and success criteria.

At the implant level, the diameter was found to influence the extent of marginal bone loss. Every 1 mm increase in implant diameter was associated with 0.08 mm of marginal bone loss. Some studies suggest that bone loss increases with short and wide implants (Winkler, Morris, and Ochi 2000; Chung et al. 2007; Monje et al. 2014). Alternatively, it has been reported that narrower implants in the posterior region exhibit greater bone loss due to reduced bone‐to‐implant contact surface area (Raikar et al. 2017). Improved stress distribution within the implant and surrounding hard tissue is seen with wider diameter implants (Allum, Tomlinson, and Joshi 2008; Song, Lee, and Shin 2017; Ding et al. 2009; Petrie and Williams 2005). A lack of clinical studies investigating implant diameter on marginal bone loss is evident. Park et al. recently performed a systematic review on narrow versus regular diameter implants for mandibular overdentures (Park, Shin, and Lee 2023). It was found that survival rate and marginal bone loss did not significantly differ between groups. Further investigation on the influence of implant diameter on success and survival criteria is needed. In particular, comparing narrow, regular, and wide‐diameter implant‐supported single crowns would be clinically relevant. The present study provided insight into the possible role that implant diameter alone may play on marginal bone loss.

A few limitations of the study do exist. Its retrospective design relies on pre‐existing data, which is assumed to be complete and accurate. The patient population was restricted to data from a single private dental office; therefore, the results may lack generalizability. Radiographic bone loss was measured using peri‐apical radiographs; therefore, only the mesial and distal aspects were accounted for. Future research may include a more in‐depth analysis including other factors such as oral hygiene habits, surgical techniques, and the inclusion of a control group. In addition, public health research should encourage investigations into implant treatment outcomes in rural populations and comparisons with those in urban populations.

## 5 Conclusion

Dental implant therapy in a rural Chinese population demonstrated high survival rates and minimal marginal bone loss. Factors such as age, location, and implant diameter influenced marginal bone loss. Future research may investigate the survival and success rates of implants in rural populations and critically assess the role diameter alone plays in marginal bone loss.

## Author Contributions

A.K., N.S., and L.L. were involved in the conception and planning of the study, data analysis, and writing the manuscript. O.Z. was in charge of data collection. All authors reviewed and approved the final version of the manuscript.

## Ethics Statement

No ethics approval was required for this private practice retrospective data collection.

## Conflicts of Interest

The authors declare no conflicts of interest.

## Data Availability

The data that support the findings of this study are available on request from the corresponding author. The data are not publicly available due to privacy or ethical restrictions.
